# A Promising Treatment Strategy for Lung Cancer: A Combination of Radiotherapy and Immunotherapy

**DOI:** 10.3390/cancers14010203

**Published:** 2021-12-31

**Authors:** Yuhei Miyasaka, Hiro Sato, Naoko Okano, Nobuteru Kubo, Hidemasa Kawamura, Tatsuya Ohno

**Affiliations:** 1 Department of Radiation Oncology, Gunma University Graduate School of Medicine, 3-39-22 Showa-Machi, Maebashi 371-8511, Japan; y.miyasaka@gunma-u.ac.jp (Y.M.); okano.n@gunma-u.ac.jp (N.O.); kubo@gunma-u.ac.jp (N.K.); kawa@gunma-u.ac.jp (H.K.); tohno@gunma-u.ac.jp (T.O.); 2 Gunma University Heavy Ion Medical Center, 3-39-22 Showa-Machi, Maebashi 371-8511, Japan

**Keywords:** lung cancer, radiotherapy, immunotherapy, immune checkpoint inhibitors

## Abstract

**Simple Summary:**

Lung cancer is a leading cause of cancer-related deaths worldwide. In the past few decades, radiotherapy has achieved outstanding technical advances and is widely used in the management of lung cancer. The anti-tumor effect of radiotherapy is mainly caused by DNA damage in cancer cells within the irradiated field. In addition, radiotherapy induces anti-tumor immune responses that are essential in cancer control. Recently, immune checkpoint molecules, such as cytotoxic T-lymphocyte-associated protein 4, programmed cell death-1/programmed death-ligand 1, and their inhibitors, have attracted significant attention for overcoming the immunosuppressive conditions in patients with cancer. Furthermore, some studies showed that the combination of immune checkpoint inhibitors and radiotherapy appears promising. In this review, we outlined evidence about the combination of radiotherapy, including particle therapy using protons and carbon ions, with immunotherapy in lung cancer treatment.

**Abstract:**

Lung cancer is a leading cause of cancer-related deaths worldwide despite advances in treatment. In the past few decades, radiotherapy has achieved outstanding technical advances and is being widely used as a definitive, prophylactic, or palliative treatment of patients with lung cancer. The anti-tumor effects of radiotherapy are considered to result in DNA damage in cancer cells. Moreover, recent evidence has demonstrated another advantage of radiotherapy: the induction of anti-tumor immune responses, which play an essential role in cancer control. In contrast, radiotherapy induces an immunosuppressive response. These conflicting reactions after radiotherapy suggest that maximizing immune response to radiotherapy by combining immunotherapy has potential to achieve more effective anti-tumor response than using each alone. Immune checkpoint molecules, such as cytotoxic T-lymphocyte-associated protein 4, programmed cell death-1/programmed death-ligand 1, and their inhibitors, have attracted significant attention for overcoming the immunosuppressive conditions in patients with cancer. Therefore, the combination of immune checkpoint inhibitors and radiotherapy is promising. Emerging preclinical and clinical studies have demonstrated the rationale for these combination strategies. In this review, we outlined evidence suggesting that combination of radiotherapy, including particle therapy using protons and carbon ions, with immunotherapy in lung cancer treatment could be a promising treatment strategy.

## 1. Introduction

Lung cancer is the most common and lethal malignancy, which accounted for approximately 11.6% of new cancer cases and 18.4% of cancer deaths in 2018 worldwide [1]. Although treatments for lung cancer continues to make progress, the 5-year relative survival is approximately 25% for non-small cell lung cancer (NSCLC), and 7% for small cell lung cancer (SCLC) [2], needing further improvements in treatments.

Radiotherapy plays a vital role in definitive, preoperative, postoperative, prophylactic, and palliative treatments for lung cancer [3,4]. In the last few decades, techniques in radiotherapy have greatly advanced, resulting in the emergence of high-precision radiotherapy modalities such as intensity-modulated radiotherapy (IMRT), stereotactic body radiotherapy (SBRT), and particle therapy using protons or carbon ions. These modalities improved cancer treatment in terms of local control, survival, and avoidance of adverse events [5]. Along with these technological improvements, controlling tumor cells outside the irradiation field is suggested to be pivotal for patients’ long-term survival.

**Figure 1:**
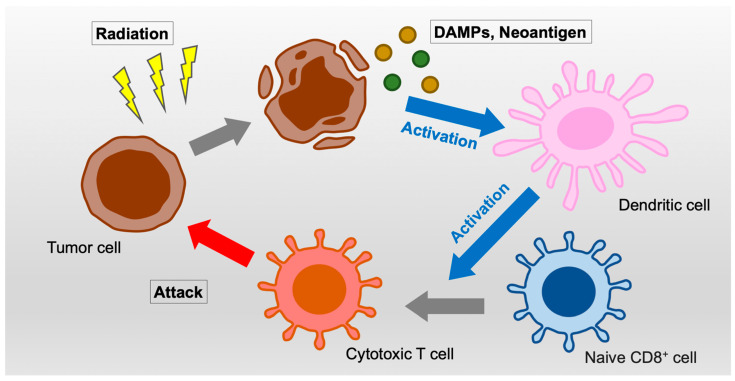
Schema of immune activation by irradiation. Abbreviation: DAMPs, damage-associated molecular patterns.

The anti-tumor effects of radiotherapy are mainly caused by deoxyribonucleic acid (DNA) damage by direct ionization and by free radicals derived from ionization of H_2_O molecules within the irradiated field. Moreover, emerging evidences have shown that radiotherapy also induces immune responses and alters the tumor microenvironment that are required for effective radiotherapy. In brief, ionizing irradiation induces immunogenic cell death, which releases damage-associated molecular patterns (DAMPs) that activate dendritic cells (DCs) and cytotoxic T lymphocytes (CTLs) (Figure 1). Simultaneously, irradiation fragmentated cytoplasmic DNA and ribonucleic acid (RNA) produce type I interferons (IFN-I) that activate systemic anti-tumor immunity [6,7,8].

The most prominent clinical response to systemic anti-tumor immunity induced by radiotherapy is the abscopal effect. This is a phenomenon in which not only irradiated tumors but also non-irradiated tumors shrink or disappear after radiotherapy. The first abscopal effect in patients with lung cancer was reported in 1983 [9]. Although the abscopal effect is rare in patients who receive radiotherapy alone, and literatures on this phenomenon were extremely limited until the last 5 years, there has been an increase in the publications on this topic as anti-tumor immune response induced by radiation has become well known. This was led by the approval and expansion of immune checkpoint inhibitors (ICIs) [10]. ICIs such as CTL-associated protein 4 (CTLA-4), programmed cell death-1 (PD-1)/programmed death-ligand 1 (PD-L1), and their inhibitors, have been known as key factors for overcoming the immunosuppressive conditions in patients with malignancies including lung cancer. Of particular note, a PD-L1 inhibitor, durvalumab, administered after chemoradiotherapy (CRT) for inoperable stage III NSCLC successfully showed survival benefits compared to CRT alone in a phase 3 randomized controlled trial (RCT) [11,12]. Furthermore, a meta-analysis showed that combinations of radiotherapy and ICIs improved survival compared to radiotherapy or ICIs alone for NSCLC (3-year OS, radiotherapy plus ICIs vs. radiotherapy alone, hazard ratio (HR), 0.82 (95% CI: 0.73–0.91); radiotherapy plus ICIs vs. ICIs alone, HR, 0.90 (95% CI: 0.82–0.99) [13]. The improvement by the combination of (chemo)radiotherapy and ICIs would be explained by inhibition of radiation-induced PD-L1 upregulation that is associated with escape from the host immune system. However, the optimal modality, dose fractionation, and timing in radiotherapy to maximize the anti-tumor efficacy is under investigation. In this review, we provided an overview of the preclinical and clinical studies and perspectives regarding the combination of radiotherapy and ICIs for lung cancer.

## 2. Rationale of a Combination of Radiotherapy with ICIs

Current evidence showed that inhibitors for immune checkpoint molecules such as CTLA-4, PD-1, and PD-L1 are beneficial in the management of various malignancies, such as lung cancer, malignant melanoma, and renal cell cancer [14]. CTLA-4 is an immune checkpoint molecule that is expressed on the surface of activated T cells and functions as a co-inhibitory receptor [15]. CTLA-4 on CTLs inactivates themselves by competitively inhibiting the binding of CD80/86 on antigen-presenting cells to CD28 on CTLs [16,17,18]. In addition, CTLA-4 on regulatory T cells suppresses immune response by binding to and downregulates of CD80/86 on dendritic cells [19,20]. PD-1 is an immune checkpoint receptor that is expressed on activated CD4-positive T cells and CD8-positive T cells [21,22]. PD-L1 is a ligand that is expressed on immune-related cells and tumor cells. PD-L1 plays a significant role in exhaustion of T cells and tumor escape from host immunity [23,24,25]. Clinical use of ICIs began with ipilimumab, a CTLA-4 inhibitor, for malignant melanoma [26]. Subsequently, the indications for ICIs were expanded to include various types of malignancies [14]. Numerous studies have evaluated the safety, efficacy, influencing factors, and cost-effectiveness of ICIs [27,28,29,30]. Combinations of ICIs and radiotherapy for lung cancer have also attracted significant attention [31,32,33].

Preclinical and translational studies have demonstrated drastic immune response to radiotherapy, which is suggested as the mechanism underlying the anti-tumor immune activation by a combination of radiotherapy and ICIs. Irradiated tumor cells present calreticulin at their surface and release DAMPs, such as adenosine-5-triphosphate (ATP) and high-mobility group protein box 1 (HMGB1) [34], that promote phagocytosis in DCs; ATP recruits DCs; HMGB1 activates DCs and CTLs. The irradiated tumor cells also activate tumor-specific CTLs via major histocompatibility complex (MHC) class I molecules and natural killer T cells via the natural killer group 2, member D (NKG2D) ligand.

Cytoplasmic DNA and RNA fragments also play an important role in tumor immune responses in the following fashion. Cyclic GMP–AMP synthase (cGAS) is a sensor of cytoplasmic DNA that triggers immune responses to microbial infections, such as viruses. cGAS mediates the production of IFN-I via the stimulator of interferon genes (STING) pathway [35]. Recent studies have shown that cGAS recognizes not only non-self but also self-derived cytoplasmic DNA fragments in cancer cells produced by irradiation-induced DNA damage. A mouse model has shown that micronuclei produced during cell division after irradiation activate the cGAS/STING pathway, resulting in a systemic immune response [6,36]. Cytoplasmic RNA is also known to promote IFN-I production [37].

Apart from the cGAS/STING pathway, IFN-I production by transcriptional RIG-I/MAVS-dependent RNA sensing and signaling has been reported as a response to radiation-induced DNA damage, especially in the case of AT-rich cytoplasmic DNA sequence production [7]. Furthermore, exosomes shuttled from irradiated cancer cells to DCs promote IFN-I production via the cGAS/STING pathway in DCs [38]. These indicate that DNA damage caused by irradiation can be a source of IFN-I release not only from irradiated cells but also from surrounding DCs. A translational study provided preclinical evidence that treatment of patients with stage I NSCLC with SBRT transformed peripheral CD8+ T cells into activated T cells and increased the production of interleukin-2 (IL-2), tumor necrosis factor-α (TNF-α), and interferon-γ (IFN-γ) but downregulated the production of transforming growth factor (TGF) -β in CD4+ T cells [39].

In contrast, radiotherapy also causes immune suppression. We previously showed that DNA damage signaling after X-ray irradiation or oxidative damage upregulated tumoral PD-L1 expression in various cancer cells [40,41]. Consistent with our preclinical data, PD-L1 expression was significantly upregulated after CRT in patients with NSCLC treated with surgery following neoadjuvant CRT [42]. Furthermore, patients with decreased PD-L1 expression after CRT showed more favorable overall survival (OS) compared with patients with unchanged or increased PD-L1 expression [43].

## 3. Radiotherapy Modalities for Lung Malignancies

Radiotherapy modalities for lung cancer presently include IMRT, SBRT, and particle therapy using protons or carbon ions. In this section, we summarize current evidence of the safety and efficacy of these modalities and potential effects on immune response.

For inoperable locally advanced lung cancer, conventional radiotherapy has been mainly used concurrently with platinum-based chemotherapy. However, in the last decade, there has been emerging evidence of the superiority of IMRT over conformal radiotherapy in the treatment of locally advanced lung cancer. In a secondary analysis of Radiation Therapy Oncology Group (RTOG) 0617, IMRT for stage III NSCLC showed lower incidence of lung toxicities with equivalent efficacy compared to conventional radiotherapy (Grade 3 or higher pneumonitis, 3.5% vs. 7.9%, respectively, *p* = 0.039) [44]. Another large-scale clinical study also showed reduction in irradiated dose to the heart in patients treated with IMRT (absolute reduction, V30 Gy, 3.0% (95% CI: 0.5–5.4); V50 Gy, 3.6% (95% CI: 2.4–4.8)) [45]. Based on these evidences, the irradiated target volume in treatment planning has been changed: elective nodal irradiation was widely utilized, while currently, involved field irradiation is recommended in some guidelines [3], although this is still under discussion. More recently, volumetric modulated arc therapy (VMAT), a novel type of IMRT in a single gantry arc that shortens the treatment time per session, has rapidly emerged [46].

SBRT is the standard definitive radiotherapy for peripheral early-stage NSCLC [3]. Previous studies have reported the superiority of SBRT, compared to conformal radiotherapy, in tumor control with tolerable toxicities [47,48,49]. Furthermore, although no robust result of phase 3 RCT exists, some studies showed that SBRT was comparable to lobectomy in patients with early-stage NSCLC [50,51,52,53,54]. SBRT for peripheral NSCLC suffers from some variations in dose fractionations [55,56,57]; therefore, to optimize it, a prospective trial that compares them is ongoing [58]. In addition to peripheral and early-stage NSCLC, more fractionated regimens for centrally located NSCLC [59] and dose escalation for T2 disease have been tested [60,61]. SBRT has also attracted attention in the management of oligometastatic malignancies. A randomized phase 2 trial of oligometastatic malignancies including lung cancer (SABR-COMET) demonstrated that SBRT was associated with favorable progression-free survival (PFS) and OS rates compared to standard care alone (5-year OS, 42.3% vs. 17.7%, respectively, *p* = 0.006; 5-year PFS, 17.3% vs. 3.2%, respectively, *p* = 0.002) [62,63]. These findings are being further investigated in a phase 3 trial (SABR-COMET-3, NCT03721341).

Particle therapy (proton or carbon-ion radiotherapy) is rapidly emerging to treat malignancies including lung cancer. A strong point of particle therapy over photon radiotherapy is sharper dose distribution derived from spread-out Bragg peak that enable to reduce irradiation dose to normal lung. For both early-stage and locally advanced NSCLC, proton beam radiotherapy (PBRT) can be applied [64]. In addition, PBRT is performed even for locoregionally recurrent NSCLC, although careful consideration of the target delineation (e.g., location, target volume, and relevant dosimetric parameters) is required to avoid significant toxicity [65,66]. Furthermore, the safety of concurrent use of chemotherapy with PBRT has been established [67]. However, the superiority of PBRT in clinical outcomes over photon radiotherapy remains controversial. A Bayesian adaptive randomization trial did not show the superiority of PBRT in terms of Grade 3 or higher radiation pneumonitis and local failure [68]. In a randomized phase 2 study comparing PBRT and SBRT, which was terminated before completion due to poor accrual, there seemed to be no difference in the prognoses [69]. On the other hand, a population-based analysis showed that PBRT was associated with favorable OS compared to non-PBRT [70]. Based on these results, a phase 3 trial is ongoing to compare PBRT and photon radiotherapy (RTOG 1308, NCT01993810). Furthermore, advances in PBRT techniques such as intensity-modulated proton therapy (IMPT) may improve clinical outcomes [71,72].

Carbon-ion radiotherapy (CIRT), external beam radiotherapy using carbon ions, is a promising radiotherapy modality because of its steeper dose distributions and higher relative biological effectiveness. Previous studies demonstrated the safety and efficacy of CIRT for early-stage [73,74,75,76], locally advanced [77,78], isolated lymph node metastasis [79], and previously irradiated NSCLC [80]. Although there is no RCT comparing CIRT and other radiotherapy modalities, we previously reported more favorable survival and local control rates after CIRT compared with that after SBRT of 48 Gy in 4 fractions to isocenter for early-stage peripheral NSCLC in a propensity score-adjusted cohort [81]. CIRT concurrent with chemotherapy for inoperable stage III NSCLC is being investigated in a prospective trial (jRCTs031190126).

Although there are a number of studies on relations between radiotherapy and immune response, the optimal dose fractionation and modality of radiotherapy to activate anti-tumor response in the clinical setting has room for investigation. Several preclinical studies revealed that relatively higher dose fractionated irradiation could lead to favorable tumor control [82,83], while hypofractionated radiation in the range of 8–12 Gy per fraction activates the cGAS/STING pathway more effectively compared with higher single doses of 20 Gy or more, in murine mammary carcinoma models [84]. Moreover, carbon-ion irradiation might influence immune response in a different manner from X-ray irradiation. In vitro and translational studies found that carbon-ion beams induced large, complex, and difficult-to-repair DNA double-strand breaks (DSBs) in irradiated cancer cells: the volume of γH2AX foci, a marker of DSBs, was 2.8-fold larger after CIRT than X-ray irradiation. Moreover, the large γH2AX foci of G2-phase cells encompassed multiple replication protein A (RPA) foci, a marker of DSBs undergoing resection during homologous recombination, which was almost never the case after X-ray irradiation [85,86]. In addition, although there was a study showing that HMGB1 release was not different between X-ray and carbon-ion irradiation with iso-survival doses [87], another study found that HMGB1 release after carbon-ion irradiation increased along with linear energy transfer (LET) in cancer cells [88]. This means that LET-modulated CIRT could be beneficial in combination with immunotherapy for malignancies. Finally, we should note that the optimal procedure of radiotherapy to activate immune response could be different by ICI because alterations in anti-tumor immunity after irradiation were different by immunity-related molecules [89].

## 4. Combination of Radiotherapy and ICIs for NSCLC

There are a large number of clinical trials with regard to combination of radiotherapy and ICIs. The ongoing phase 3 clinical trials on radiotherapy with PD-1 or PD-L1 inhibitors for NSCLC are summarized in Table 1. Of importance, a meta-analysis showed that the incidence of severe treatment-related adverse events after a concurrent combination of anti-PD-1/PD-L1 therapies with radiotherapy was 12.4%, while those with chemotherapy and targeted therapy were 68.3% and 35.9%, respectively [90]. Thus, radiotherapy might be safer compared to systemic therapies in combination with ICIs.

Durvalumab, a PD-L1 inhibitor, as an adjuvant treatment following definitive CRT for unresectable stage III NSCLC, has the most robust evidence of survival benefits among combination therapies of radiotherapy and immunotherapy for lung cancer. A phase 3 RCT (PACIFIC trial) showed significantly favorable PFS and OS rates in patients who received CRT followed by durvalumab compared to those who received CRT alone (18-month PFS rate: 44.2% vs. 27.0%, respectively, HR, 0.52 (95% confidence interval (CI), 0.42–0.65), *p* < 0.001; 24-month OS rate: 66.3% vs. 55.6%, respectively, HR, 0.68 (95% CI: 0.47–0.997), *p* = 0.0025) with comparable Grade 3 or higher adverse events (29.9% vs. 26.1%, respectively), including pneumonia (4.4% vs. 3.8%, respectively) and pneumonitis or radiation pneumonitis (3.4% vs. 2.6%, respectively) [11,12]. A post hoc analysis of the PACIFIC trial revealed consistent improvements with longer follow-up (4-year OS rate, 49.6% vs. 36.3%, respectively, HR, 0.71 (95% CI: 0.57–0.88); 4-year PFS rate, 35.3% vs. 19.5%, respectively, HR, 0.55 (95% CI: 0.44–0.67)) [91]. The results of this trial have changed the standard therapies for unresectable stage III NSCLC, although it should be noted that patients with progression and/or Grade 2 or higher radiation pneumonia after CRT were excluded from the indication of adjuvant durvalumab. Thus, developing a treatment strategy for such a patient is a future challenge. Several sub-analyses of the PACIFIC trial provided intriguing results. The administration of durvalumab contributed to favorable PFS among all subgroups stratified by tumoral PD-L1 expression levels [92]. These results might be affected by tumoral PD-L1 upregulation induced by radiotherapy since a meta-analysis showed that prognostic benefit of ICIs for NSCLC was not observed in patients with PD-L1 expression <1% [93]. Another sub-analysis found that the PFS improvement in the patients who received durvalumab after CRT was more pronounced among patients who completed their radiotherapy course within 14 days before randomization (HR, 0.39 (95% CI: 0.26–0.58)) compared to those who finished their radiotherapy sooner (HR, 0.63 (95% CI: 0.49–0.80)) [94]. These results suggest the combination of CRT and durvalumab is a remarkable treatment strategy. Furthermore, durvalumab is being investigated in clinical trials as concurrent and consolidative therapy with CRT for stage III NSCLC (NCT03519971, NCT04092283), consolidative therapy after SBRT for lymph node-negative stage I/II NSCLC (NCT03833154), consolidative therapy after sequential or concurrent CRT for stage III NSCLC (NCT03706690), consolidative therapy after sequential CRT (NCT03693300), and concurrent CRT for stage III NSCLC in the real world.

Atezolizumab, another PD-L1 inhibitor, has also been investigated for combination with radiotherapy in clinical trials. A phase 2 trial of CRT (including PBRT; median radiation dose was 66 Gy) plus atezolizumab for NSCLC demonstrated that their concurrent and maintenance use presented acceptable toxicities and favorable survival. This trial was consisting of CRT followed by consolidation/maintenance atezolizumab group and CRT concurrently with atezolizumab followed by the consolidation/maintenance atezolizumab group. Importantly, the authors concluded that atezolizumab with concurrent CRT is feasible, with similar rates in both groups (all adverse events of Grade 3 or higher: 80% vs. 80%; immune-related adverse events of Grade 3 or higher: 30% vs. 20%, and pneumonitis of Grade 2 or higher: 10% vs. 16%, respectively). In this study, prognoses were not different according to PD-L1 status (1-year PFS rates: PD-L1 IHC ≥1% vs. <1%, 70% vs. 50%, respectively, HR, 2.0 (95% CI: 0.742–5.547); PD-L1 IHC ≥50% vs. <50%, 70% vs. 58%, respectively, HR, 2.6 (95% CI: 0.858–7.802)) [95], as same as the results of the PACIFIC trial [93]. Of note, there is an ongoing phase 3 trial of atezolizumab plus T cell immunoreceptors with immunoglobulin and immunoreceptor tyrosine-based inhibitory motif (ITIM) domains (TIGIT) inhibitor, tiragolumab vs. durvalumab after CRT. Such a mixed use of ICIs might benefit in combination with radiotherapy [96].

Pembrolizumab is a PD-1 inhibitor that is widely used in the management of various malignancies including lung cancer. A secondary analysis of a prospective trial (KEYNOTE 001) demonstrated that favorable PFS and OS after receiving pembrolizumab were observed in patients who previously received radiotherapy (median PFS, 4.4 months vs. 2.1 months, HR, 0.56 (95% CI: 0.34–0.91), *p* = 0.019; median OS, 10.7 months vs. 5.3 months, HR, 0.58 (95% CI: 0.36–0.94), *p* = 0.026,) [97]. In this analysis, patients with a history of radiotherapy did not have a higher rate of Grade 3 or higher pulmonary toxicity compared to those without a history of radiotherapy (*p* = 0.440). Similar results have been reported in other retrospective studies [98,99]. A phase 2, multi-institutional RCT (PEMBRO-RT) presented trends of favorable prognoses in patients who received pembrolizumab after radiotherapy for advanced NSCLC compared to those who received pembrolizumab alone (median PFS, 6.6 vs. 1.9 months, respectively, HR, 0.71 (95% CI: 0.42–1.18), *p* = 0.19; median OS, 15.9 vs. 7.6 months, respectively, HR, 0.66 (95% CI: 0.37–1.18), *p* = 0.16) [100], whereas in a phase 1/2, single-institutional, RCT (MDACC), a weaker trend was observed (median PFS, 9.1 vs. 5.1 months, *p* = 0.52) [101]. These studies could not demonstrate a significant superiority of pembrolizumab plus radiotherapy over pembrolizumab alone; however, a pooled analysis of these studies showed favorable PFS and OS rates for pembrolizumab plus radiotherapy compared to those for pembrolizumab monotherapy (median PFS, 9.0 vs. 4.4 months, respectively, HR, 0.67 (95% CI: 0.45–0.99), *p* = 0.045; median OS, 19.2 vs. 8.2, respectively, HR, 0.67 (95% CI: 0.54–0.84), *p* = 0.0004) [102]. This pooled analysis showed that the rate of pembrolizumab-related adverse events was comparable to those of other pembrolizumab studies. It should be noted that radiotherapy regimens and timings were different among these trials, that is, pembrolizumab was administered after completion of SBRT of 24 Gy in 3 fractions in PEMBRO-RT, while it was administered concurrently with SBRT of 50 Gy in 4 fractions or conventional radiotherapy of 45 Gy in 15 fractions in MDACC. Interestingly, in MDACC, overall response rate out of the field tended to be higher for SBRT plus pembrolizumab compared to in conventional radiotherapy plus pembrolizumab (38% vs. 10%, respectively, *p* = 0.11) [101], and a pooled analysis including the MDACC study showed that the abscopal response rate was higher in the SBRT group compared to in the traditional radiotherapy group (*p* < 0.0001) [103], suggesting that differences in radiation doses, fractionations, and modalities have the potential to affect clinical outcomes. For oligometastatic NSCLC, a phase 2 study showed that locally ablative therapy followed by pembrolizumab was associated with favorable PFS compared with a historical control of radical treatments (PFS, median, 19.1 vs. 6.6 months, respectively, *p* = 0.005) [104,105]. In addition, adjuvant pembrolizumab following CRT for inoperable stage III NSCLC showed a 2-year OS rate of 62.0% with tolerable toxicities (symptomatic pneumonitis: 17.2% (Grade 3, 4.3%; Grade 4, 1.1%; Grade 5, 1.1%)) [106].

Nivolumab has also been investigated in combination with radiotherapy. A phase 2 trial of the concurrent CRT plus nivolumab for stage III NSCLC provided tolerable safety profiles including no Grade 3 or higher pneumonitis reported 3 months after CRT [107]. The efficacy of this combination was also provided in the expanded cohort (median PFS, 12.7 months; median OS, 38.8 months) [108]. A larger RCT of nivolumab plus ipilimumab concurrent with CRT for stage III NSCLC is ongoing (NCT04026412). For stage IV NSCLC, adding of SBRT to nivolumab is being tested in a phase 2 RCT (NIVORAD, ACTRN12616000352404). For recurrent NSCLC after CRT, a retrospective study provided an interesting result: patients who had a shorter interval from the CRT to initiation of salvage anti-PD-1 therapy tended to have favorable PFS compared to those who had the longer interval (median, 17 months (95% CI: 0.47–not reached) vs. 4.9 months (95% CI: 1.47–8.43), respectively) [109]. This might suggest that alterations in tumor characteristics and host immunity after CRT could lead to better outcomes with salvage anti-PD-1 therapy.

Other ICIs, such as TIGIT [110,111,112,113], lymphocyte activation gene-3 (LAG-3) [114], and T cell immunoglobulin and mucin domain 3 (TIM-3) [115], are under development. Of note, tiragolumab, a TIGIT inhibitor, has being investigated in a phase 3 trial of testing adjuvant use with atezolizumab after CRT for stage III NSCLC (NCT04513925).

## 5. Combination of Radiotherapy and ICIs for SCLC

Definitive radiotherapy for SCLC is mainly utilized for limited-stage disease with concurrent chemotherapy. The standard radiotherapy regimen for limited-stage SCLC is accelerated hyperfractionated radiotherapy (AHF), which involves irradiation with 45 Gy in 30 fractions, twice daily [116,117], although a higher dose AHF with 60 Gy in 40 fractions was promising in a phase 2 RCT (median OS: 60 Gy vs. 40 Gy, 37.2 months (95% CI: 28.4–46.1) vs. 22.6 months (95% CI: 17.1–28.1), respectively, HR, 0.61 (95% CI: 0.41–0.90), *p* = 0.012, with no significant difference in toxicity) [118]. Regarding radiotherapy modalities for SCLC, conformal X-ray radiotherapy is generally used, and moreover, PBRT is being considered as a radiotherapy treatment option [119,120]. After the definitive treatment, prophylactic cranial irradiation (PCI) was also considered.

The addition of ICIs to CRT for limited-stage SCLC has been investigated in several clinical trials. A phase 1/2 trial of concurrent CRT and pembrolizumab showed safety (symptomatic pneumonitis, 15%; one dose-limiting toxicity) and favorable survival outcomes (median OS, 39.5 months; median PFS, 19.7 months) [121], which were better than those of AHF with 45 Gy in 30 fractions in the previous trial (median OS, 30 months; median PFS, 15.4 months) [117]. However, it should be noted that a phase 2 trial of nivolumab plus ipilimumab following CRT and PCI (STIMULI (NCT02768558)) did not show significant improvement either in PFS or in OS in the experimental arm compared to in the observation arm (median PFS, 10.7 vs. 14.5 months, respectively, HR, 1.02 (95% CI: 0.66–1.58); median OS, not reached vs. 32.1 months, respectively, HR, 0.95 (95% CI: 0.59–1.52)) with 62% of Grade 3 or higher adverse events vs. 25%, respectively. The authors in the study claimed that the efficacy results might be affected by a short period on active treatment related to toxicity and treatment discontinuation [122]. Reconsideration of the combination and/or procedures might lead to different results.

Ongoing phase 3 clinical trials are summarized in Table 2. Durvalumab after CRT is being investigated with or without a CTLA-4 inhibitor, tremelimumab (ADRIATIC study) [123]. Atezolizumab is being tested as a concurrent treatment (NRG-LU005, NCT03811002, phase 2/3) with CRT as well as adjuvant treatment (ACHILES trial, NCT03540420, phase 2). In addition to LS SCLC, addition of radiotherapy to atezolizumab for ES SCLC is being tested in a phase 2/3 trial (RAPTOR, NCT04402788). Pembrolizumab is being investigated as combination therapy with CRT plus olaparib, a poly ADP-ribose polymerase (PARP) inhibitor.

## 6. Conclusions and Future Direction

Radiotherapy techniques have greatly advanced in just a few decades. The advent of ICIs has changed treatments for lung cancer. Furthermore, combination of radiotherapy and ICIs seemed to be promising in preclinical and clinical studies. In vitro studies showed X-ray irradiation promoted PD-L1 expression in cancer cells, by which cancer cells may escape from a host immunity. In the clinic, the PACIFIC trial provided the robust evidence of prolonged survival of locally advanced NSCLC after CRT followed by the PD-L1 inhibitor, durvalumab with acceptable toxicity. Combined radiotherapy and ICIs seemed to be also indicated for metastatic NSCLC. Even some patients with early-stage NSCLC may benefit from ICIs after SBRT or particle therapy. Additionally, combining CRT with ICIs may prevent distant metastasis not only in limited-stage SCLC but also in extensive-stage SCLC.

However, optimization in the combination therapy is still required. For instance, although the PACIFIC trial provided the robust evidence of safety and efficacy, appropriate dose restrictions in radiotherapy when followed by durvalumab have not been established because there is no detailed data in the PACIFIC trial with regard to radiotherapy regimens, such as dose fractionation and dose-volume histogram parameters. The timing of the addition of ICIs to radiotherapy also remains to be optimized. The sub-analysis of the PACIFIC trial showed that shorter periods between CRT and durvalumab might contribute to favorable outcomes, but preclinical studies suggested that the optimal timing of the use of radiotherapy and ICIs could differ according to the type of ICI. In addition, as clinical investigation for other immune checkpoint molecules such as TIGIT, LAG-3, and TIM-3 is underway, the key molecules and kinetics that influence oncologic outcomes need to be more explored.

## Figures and Tables

**Table 1:** Summary of ongoing phase 3 trials of immunotherapy combined with chemoradiotherapy for lung cancer (ClinicalTrials.gov, accessed on 27 April 2021).

NCT Number	Trial	Stage	Medication	Treatment
NCT03391869	LONESTAR	IV	Nivolumab + Ipilimumab	RT Surgery Obs.
NCT03519971	PACIFIC-2	III	Durvalumab Placebo	CRT
NCT03833154	PACIFIC-4	T1-3N0M0	Durvalumab Placebo	SBRT
NCT03774732	NIRVANA-LUNG	IIIB-IV	Pembrolizumab	CRT CT
NCT03867175		IV	Pembrolizumab	SBRT Obs.
NCT03924869	MK-3475-867 KEYNOTE-867	I-IIA	Pembrolizumab Placebo	SBRT
NCT04214262		T1-3N0M0	Atezolizumab Placebo	SBRT
NCT04092283		III	Durvalumab	CRT
NCT04380636	MK-7339-012 KEYLYNK-012	III	Pembrolizumab + Olaparib Durvalumab	CRT
NCT04465968	DEEP_OCEAN	SST	Durvalumab	CRT
NCT04597671	NVALT28	III, treated	Durvalumab	PCI Obs.
NCT04026412	CheckMate 73L	III	Nivolumab + Ipilimumab Placebo	CRT
NCT04513925	SKYSCRAPER-03	III	Durvalumab Atezolizumab + Tiragolumab	CRT

Abbreviations: RT, radiotherapy; Obs., observation; CRT, chemoradiotherapy; SBRT, stereotactic body radiotherapy; CT, chemotherapy; SST, superior sulcus tumor; PCI, prophylactic cranial irradiation.

**Table 2:** Summary of ongoing phase 3 trials of immunotherapy combined with chemoradiotherapy for small cell lung cancer (ClinicalTrials.gov, accessed on 27 April 2021).

NCT Number	Trial	Stage	Medication	Treatment
NCT03811002	NRG-LU005	LS	Atezolizumab Placebo	CRT
NCT04402788	NRG-LU007 RAPTOR	ES	Atezolizumab	RT Obs.
NCT04624204	MK 7339-013 KEYLYNK-013	LS	Pembrolizumab + Olaparib	CRT + PCI
NCT03703297	ADRIATIC	LS	Durvalumab + Tremelimumab	CRT

Abbreviations: LS, limited-stage; ES, extensive stage; CRT, chemoradiotherapy; Obs., observation; PCI, prophylactic cranial irradiation.
